# *Botryosphaeria* Dieback (*Lasiodiplodia viticola*): An Imminent Emerging Threat to the Moroccan Vineyards

**DOI:** 10.3390/plants11162167

**Published:** 2022-08-21

**Authors:** Jihane Kenfaoui, Rachid Lahlali, Mohammed Mennani, Nabil Radouane, Khadija Goura, Hajar El Hamss, Lahsen El Ghadraoui, Florence Fontaine, Abdessalem Tahiri, Essaid Ait Barka, Said Amiri

**Affiliations:** 1 Phytopathology Unit, Department of Plant Protection, Ecole Nationale d’Agriculture de Meknès, Km10, Rte Haj Kaddour, BP S/40, Meknes 50001, Morocco; 2 Laboratory of Functional Ecology and Environmental Engineering, Sidi Mohamed Ben Abdellah University, P.O. Box 2202, Route d’Imouzzer, Fez 30500, Morocco; 3 Laboratory of Biotechnology and Valorisation of Biological Resources, Department of Biology, Faculty of Science, Moulay Ismail University, Meknes 50070, Morocco; 4 Unité de Recherche Résistance Induite et Bio-Protection des Plantes-EA 4707 USC INRAE 1488, Université de Reims Champagne-Ardenne, 51100 Reims, France

**Keywords:** grapevine trunk diseases, *Botryosphaeria* dieback, phytosanitary problems, identification, pathogenicity

## Abstract

A decline of various grapevines (*Vitis vinifera* L.) in the province of Doukkala in Morocco was observed in 2021. The causal pathogen was identified as *Lasiodiplodia viticola* based on morphological characteristics and phylogenetic analysis of the internal transcribed region (ITS), the β-tubulin gene (TUB) and calmodulin (cmdA). Koch’s postulates were confirmed by successful re-isolation of *L. viticola* from plants inoculated with the pathogen under controlled conditions. The disease was shown to be prevalent in Bni Hilal (71.43%), Laamria (60%), and Boulaouane (40%) districts, but was quasi-absent in Lmechrek. To understand the dominance of *L. viticola* as one of the grapevine trunk pathogens, effects of temperature (10–40 °C) and pH (pH 3–pH 12) on growth and sporulation were investigated. The species were able to grow in a range of temperatures ranging from 15 to 40°C and showed a higher growth rate at 35 °C. The fungus were also characterized by a broad optimum pH ranging between 3–12. This study is the first report dealing with *L. viticola* associated with grapevine trunk diseases in Morocco. Additional studies are therefore required to understand the high occurrence of this disease in vineyards, which is likely due to climate changes. A good understanding of this complex disease might help to develop a reliable and sustainable preventive control strategy.

## 1. Introduction

*Vitis vinifera* L., the grapevine or cultivated grapevine, is a dicotyledonous angiosperm plant of the *Vitaceae* family [1]. It is one of the most widely cultivated fruit plants worldwide. Grapevines are cultivated on all continents except Antarctica. Overall, in 2020, the world area of grapevines was 7.3 million hectares, which includes the total surface area planted for all purposes (wine and juice, table grapes and raisins) and young grapevines not yet in production [2]. In Morocco, the culture of vines occupies an area of more than 50,000 hectares, with an annual production of about 452,000 tons [3].

Despite the importance of the vine in some regions in Morocco and worldwide, the production of grapes is not always guaranteed, due to several problems and threats. Among these problems, those related to the phytosanitary state of the vine are the most serious. One of the major problems of grapevine cultivation is its susceptibility to a wide range of microorganisms such as viruses, bacteria, nematodes and fungi [4]. The most commonly known fungal diseases are downy mildew (*Plasmopara viticola*), powdery mildew (*Erysiphe necator*) and botrytis (*Botrytis cinerea*). These can attack different herbaceous parts of the vine such as the leaves, stems and berries. Nevertheless, the wood of the vine is also colonized by a wide range of vascular fungi, some of which are phytopathogenic [5]. Grapevine trunk diseases (GTD) are the most widespread fungal diseases, harming grapevines in all of the world’s major growing regions, and there is no way to fully eradicate them [6,7]. GTDs are caused by 133 fungal species belonging to 34 different genera and 9 different families, with similar life cycles and epidemiology [6,7]. These pathogens cause damage in vineyard with various levels of severity, ranging from simple reactions to severe symptoms [8]. Diseases affecting wood, commonly known as grapevine trunk diseases (GTD), are currently at the forefront of winegrowers’ concerns, not only in Morocco, but around the world [9]. The most common ones are Esca disease, *Phomopsis*, Eutypiosis and *Botryosphaeria* diebacks. They are therefore very damaging to the sustainability of the vinicultural heritage. These wood-inhabiting fungi live in and colonize the wood of the perennial organs, impeding water transport in plants by clogging the xylem vessels and, consequently, decreasing their adsorption capacity of water and nutrients [10,11]. However, their effect can be undetectable for years due to the slow growth of the pathogens in the vascular tissues, which enables the visibility of symptoms on the canopy [12]. They can be characterized by more-or-less severe forms, going up to apoplexy, or by slow forms leading to the progressive weakening of the plant (loss of vigor) ending in the death of one of its parts (spurs, arms) and then its totality. In general, at the time of the appearance of leaf symptoms, the disease has already developed to a severe situation, which can lead, in some cases, to the death of a cordon or of the entire plant [13].

*Botryosphaeria* dieback is caused by several pathogens, primarily *Neofusicoccum parvum* (Pennycook and Samuels) Crous, Slippers & Phillips, *Diplodia seriata* de Notaris, *Botryosphaeria stevensii* (Fries) Montagne and *Botryosphaeria dothidea* (Mougeot) Cesati and de Notaris. To date, 26 *Botryosphaeriaceae* species from the genera *Botryosphaeria*, *Diplodia*, *Dothiorella*, *Lasiodiplodia*, *Neofusicoccum*, *Sphaeropsis* and *Spencermartinsia* have been associated with grapevine diebacks [6,14]. Very little is currently known about the epidemiology of *Botryosphaeria* diseases of grapevines [15,16], especially in Morocco. The increasing number of *Botryosphaeria* species found on grapevines makes epidemiological research on this pathogen more difficult. Further, species of *Botryosphaeriaceae* can differ in their epidemiology, symptoms and relative importance [5]. Each year, they lead to the death of infected vines, forcing winegrowers to uproot the majority of the vines in their vineyards. As a result, more disease-resistant vines must be planted, and this can lead to the loss of the typicity and quality of a particular region’s wine [9], which causes important economic losses, reducing yield, quality and longevity of the vineyards [12,17,18].

**Figure 1:**
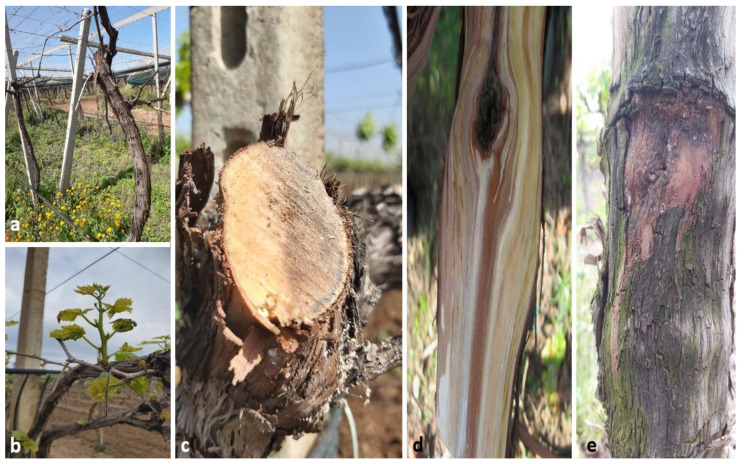
Infected grapevines displaying several symptoms in different Doukkala vineyards. (**a**) Typical dieback of grapevines; (**b**) grapevine showing overall stunting with short shoot internodes; (**c**) characteristic necrosis of *Botryosphaeria* dieback; (**d**) bark peeling with a brown strip and (**e**) symptoms of canker, reddish-brown with well-demarcated elliptical areas on the outer surface of the vine trunk.

In March 2021, grapevine trunks in vineyards in Doukkala, Morocco showed bark peeling with a brown strip a few centimeters wide, starting from the twig to reach the rootstock; the cross-sections manifested characteristic necrosis of *Botryosphaeria* diebacks (Figure 1). The disease further progressed to produce diebacks of entire stems and the death of plants. The main aim of the present study was to identify and characterize the causal agent(s) of this disease using microbial and molecular analysis. Pathogenicity tests were then carried out to validate the capacity to produce dieback symptoms.

## 2. Results

### 2.1. Species Identification and Morphological Features of Isolates

**Figure 2:**
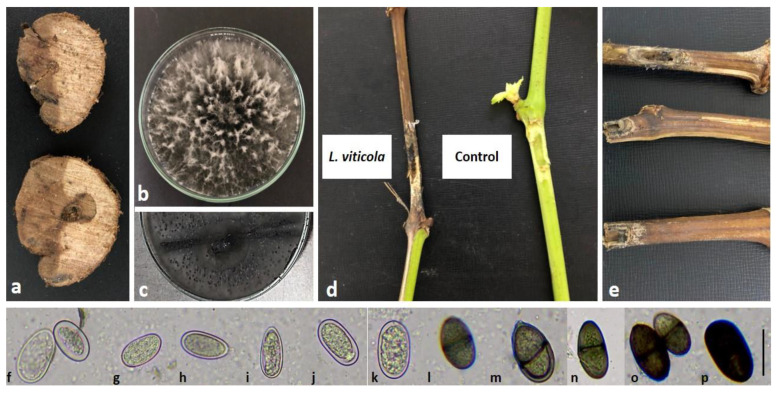
*Lasiodiplodia viticola* causing *Botryosphaeria* dieback on the grapevine. (**a**) Characteristic necrosis of *Botryosphaeria* dieback; (**b**) pure *L. viticola* culture on a 9 cm diam plate containing potato dextrose-agar (4 days); (**c**) pycnidia formed on pine needles (2 weeks); (**d**,**e**) pathogenicity of *L. viticola* on grapevine shoots (left: shoot inoculated with the fungus, right: control); (**f**–**k**) aseptate, hyaline and granulated conidia; (**l**–**n**) oneseptate conidia and (**o**,**p**) mature brown conidia with longitudinal striations. Bars = 20 µm.

The trunk isolated from symptomatic grapevine plants that were sampled in the studied vineyard in Doukkala province was consistently found with mycelial features of *Botryosphaeriaceae* (Figure 2a). One representative isolate amongst ten similar ones obtained from the symptomatic sampled wood was characterized.

Fungal colonies were fast-growing on PDA medium (7.98 cm diameters in 7 days), raised and blackish with abundant sporulation and undulate margins after two weeks of inoculation on PDA containing pine needles (Figure 2b,c). One representative culture was been deposited in the PhytoENA-Meknès-Microorganisms Collection (CMENA) under the accession number CMENA03-1. Conidia were brown and oval with rounded and pointed ends. Immature conidia were hyaline, thick-walled and aseptate, and some of them were densely granulated. When mature, conidia were brown with longitudinal striations and one septum, and measured about 24.52 ± 1.89 × 12.68 ± 0.30 µm (Figure 2f–p). This morphological criterion is known as a typical trait of the *Botryosphaeriaceae* species as described by Slippers and Wingfield [19].

### 2.2. Pathogenicity Test

The isolates from grapevine trunks proved to be pathogenic on grapevine-detached shoots cv. *Vitis vinifera*, and showed disease symptoms 15 days post-inoculation. All 10 shoots used in the experiment showed symptoms similar to those observed in the field. The bark tissue turned brown and rotted, and the lesion reached the wood and subsequently led to the death of the whole detached branches within four weeks post-inoculation (Figure 2e). 

The average lesion length recorded was 9.70 cm (*p* < 0.0001). Conversely, control cuttings treated with PDA plugs only had an average lesion length of 0.6 cm (Figure 2d). Statistical analysis revealed significant differences in lesion length between the control and young shoots inoculated with the fungus. The pathogenic fungus sporulated on the necrotic tissues and conidia were transferred to PDA plates to produce identical colonies, fulfilling Koch’s postulates. The identity of an isolate obtained from necrotic wood of symptomatic, artificially inoculated cuttings was determined according to colony morphology, microscopic observations and ITS rDNA sequencing.

### 2.3. Molecular Identification

The sequences of ITS, β-tubulin and cmdA, were edited and aligned using BioEdit software version 7.0.5.3 (Raleigh, NC, USA) and then deposited in GenBank under the accession numbers OK087687, OL348063 and ON109769, respectively. The sequence analysis confirmed the morphological identification of the isolate.

**Figure 3:**
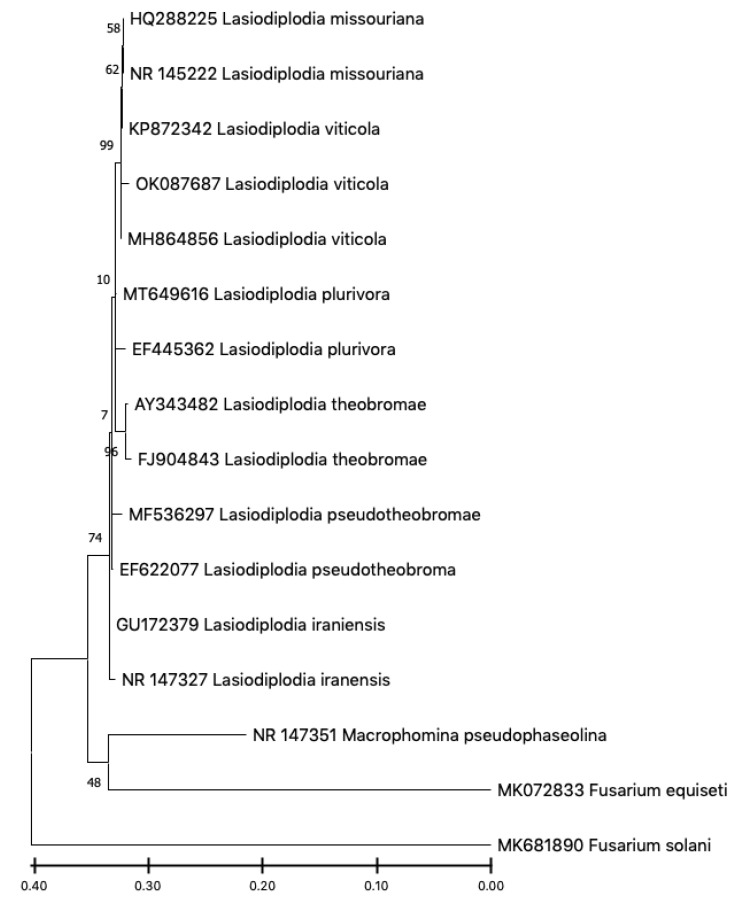
Phylogenetic tree of *Lasiodiplodia viticola* constructed using the maximum likelihood method with 1000 bootstrap values, based on their internal transcribed spacers (ITS), β-tubulin and calmodulin (cmdA) sequences, showing the position of our fungus clustered with its reference.

The resulting sequence shared 99.4% similarity based on the ITS gene, with isolates of *Lasiodiplodia viticola* as described on Myrtales in Southern Africa (MH864856). However, 100% similarity was found during the Blast search of the sequence of the β-tubulin and cmdA genes on *Vitis vinifera* L. in France (KP699091) and the USA (KU886794), respectively. This identification was further confirmed by phylogenetic analysis of concatenated sequence alignment of the three genes using MEGA 11 version 11.0.8 (Philadelphia, PA, USA) (Figure 3). The evolutionary history was inferred by using the maximum likelihood method and Kimura 2-parameter model with 1000 bootstrap values.

### 2.4. Distribution of the Pathogenic Fungus in the Surveyed Vineyards

The prevalence of the fungus in samples taken from each district is shown in Table 1. Results indicate that the majority of samples were under the attack of *L. viticola* (50%). Moreover, no samples from the Lmechrek district were positive for the pathogen. However, the Bni Hilal location was the most infected of all the surveyed vineyards in the Doukkala-Abda region.

### 2.5. Physiological Traits

#### 2.5.1. Effect of Temperature on Radial Growth Rate

**Figure 4:**
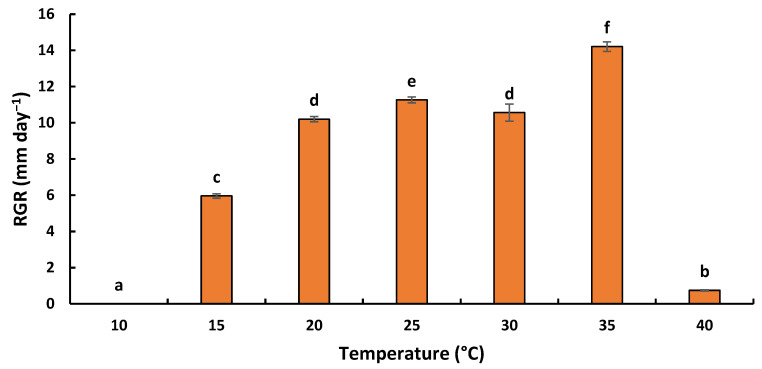
Radial growth rate (mm day^−1^) versus temperature (°C) for *L. viticola*. Datasets are the mean of three replicates. Error bars represented standard deviations. Treatments with the same letter are not significantly different according to the Tukey test *p* ≤ 0.05.

The effect of temperature was evaluated within a range of 10 to 40 °C. The results obtained highlighted the substantial effect of incubation temperature on the growth of *L. viticola* (Figure 4). Temperature had a significant effect on radial growth rates (RGR) (F: 1779.73; ddl: 6; *p* < 0.001). The species was able to grow only in a range of temperatures between 15 and 40 °C. At the low extremes, colony growth after 7 days was negligible for all fungi at 5 °C and 10 °C. *L. viticola* showed a higher growth rate at 35 °C, with a radial growth rate of 14.21 ± 0.26 mm⋅day^−1^. 

#### 2.5.2. Effect of pH on Radial Growth Rate

**Figure 5:**
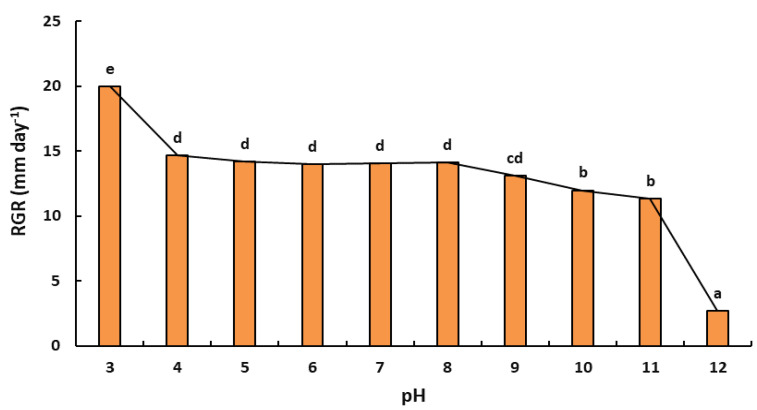
Plots of colony growth rate (mm day^−1^) versus pH for *L. viticola*. Datasets are the mean of three replicates. Error bars represented standard deviations. Treatments with the same letter are not significantly different according to the Tukey test *p* ≤ 0.05.

The effect of pH was evaluated within a range of 3 to 12. The results obtained highlighted the substantial effect of pH on the growth of *L. viticola*. The radial growth rate was been shown to decrease with increasing pH values (Figure 5). The pH had a significant effect on radial growth rates (F: 130.72; ddl: 9; *p* < 0.001). With an optimal growth with pH 3 to 8, *L. viticola* was less demanding concerning acidity and showed a higher growth rate at pH 3, with a radial growth rate of 20.00 ± 0.29 mm·day^−1^. The fungus was tolerant to alkaline conditions, as growth only slightly diminished at a pH ranging between 8 to 11, whilst pH reaching 12 almost inhibited the growth of the studied fungus, with the lowest growth rate reaching 2.71 ± 0.29 mm·day^−1^.

## 3. Discussion

This study confirmed for the first time the occurrence of *Botryosphaeria* dieback, one of the main GTDs affecting young and mature vines in Morocco. Moreover, this study represents the first attempt to characterize the impact of two important environmental factors, temperature and pH, on the ecology of the most prevalent fungus in the studied region.

In recent years, symptoms of decline have been observed on grapevines in the Doukkala-Abda region, causing serious damages and reducing overall yield. In this study, we confirmed through morphological and molecular characterization and pathogenicity tests that these symptoms of decline were mainly due to the presence of *L. viticola*. This species, which threatens most of the Moroccan vineyards, was found in 10 out of 20 samples from different sites in the Doukkala-Abda region. Symptoms observed in the field, as well as those obtained from the pathogenicity test of this study, revealed a substantial similarity to those of dieback diseases. Furthermore, under a light microscope, conidia characteristics of *L. viticola* almost matched the size given for this specie in former research (22.90 ± 2.05 × 10.70 ± 0.20) [17,18]. The pathogenicity tests carried out in the same study highlighted that isolates of *Lasiodiplodia missouriana*, *L. viticola* and *Neofusicoccum ribis* Crous, Slippers and A.J.L. Phillips were the most virulent species on *Vitis vinifera* L. cultivars compared with the other studied fungal species [18]. According to a previous study, *Lasiodiplodia viticola* was described from grape cultivars and has also been found on *Mangifera indica* Wall. in Brazil. Based on tub2, cmdA, ITS and rpb2 sequence analysis, *L. viticola* groups were found with *L. mediterranea* and hybrid species *L. missouriana* [12]. This study suggested that isolates of *L. viticola* have probably arisen from hybridization between these two species [19]. Moreover, based on tef1-α sequences, it was demonstrated that *L. viticola* is identical to the hybrid species *L. Brasiliense*, which is closely related to *L. theobromae*. Consequently, it was concluded that *L. viticola* has probably arisen from hybridization between *L. mediterranea* and *L. theobromae*, since the grapevine is a known host of those two species [17,19]. This is in agreement with the analysis of the phylogenetic tree in this study, which underlined the strong similarity of our isolates with *L. missouriana* and *L. theobromae* mentioned above. Based on literature reports, the pathogenic fungus was described in grapevine cultivars [18] and has also been found on mango in Brazil [20]. In the French vineyards, this fungus was reported in 2016 and is associated with other fungi, namely *Diplodia intermedia* and *Spencermartinsia viticola* A.J.L. Phillips and J. Luque [21].

Although trunk diseases are a major threat for grapevines production, only limited information about the ecology of the main pathogens involved has been published so far. To our knowledge, this is the first study dealing with the impact on the ecology of *L. viticola* associated with grapevine trunk diseases. In the present work, we evaluated the effects of ecophysiological factors, mainly temperature (10–40 °C) and pH (3–12) and their interaction on radial growth rate (g). The present study confirmed that the environmental factors significantly affected the radial growth rate of the evaluated fungus. Our results underline that the maximum radial growth rates were obtained at 25–35 °C. The results from this study corroborated those of a study on the mycelial growth of *L. theobromae*, which was capable of growing at temperatures ranging between 8–36 °C with an optimal radial growth at 28 °C [22]. Hence, these results confirmed that attention should be paid to temperatures above 25 °C to control the growth of these species and to avoid any risk of infection by these hot climate pathogens. As for the pH experiment, *L. viticola* was able to grow within a wide range of pH from 3 to 8. The fungus, however, almost failed to grow in an alkaline environment. The result indicated that a slightly acidic pH to neutral pH was optimum for the growth of *L. viticola*. This study of the influence of temperature and pH on the growth of *L. viticola* may be useful to determine whether changes in environmental conditions are the cause of its occurrence in recent years. Úrbez-Torres highlighted the importance of understanding the epidemiology of *Botryosphaeriaceae* species by studying sources of inoculum, conditions that endorse spore release, seasonal spore release patterns, seasonal susceptibility of pruning wounds and factors that favor infection [23]. According to research, various species require different climatic conditions to create fruiting structures. As a result, *Botryosphaeria* infection occurs in a variety of climatic conditions [24]. Moreover, the longer the period of wetness and high relative humidity extends, the more spores are produced and released, hence creating a much higher inoculum load and increasing the severity of infection [25]. According to several authors, an increase in wetness duration combined with high inoculum levels led to an increase in severe pistachio and peach tree infections [26,27].

*Lasiodiplodia viticola* is a polyphagous pathogen whose host range comprises several plant species worldwide, as described above. To our knowledge, this is the first report of *L. viticola* causing *Botryosphaeria* dieback of grapevine in Morocco. This result highlights the gaps in knowledge of the pathogen in Morocco, providing early warnings of new or unreported diseases and fungi that may threaten the Moroccan viticulture industry.

## 4. Material and Methods

### 4.1. Survey and Sampling Sites

**Figure 6:**
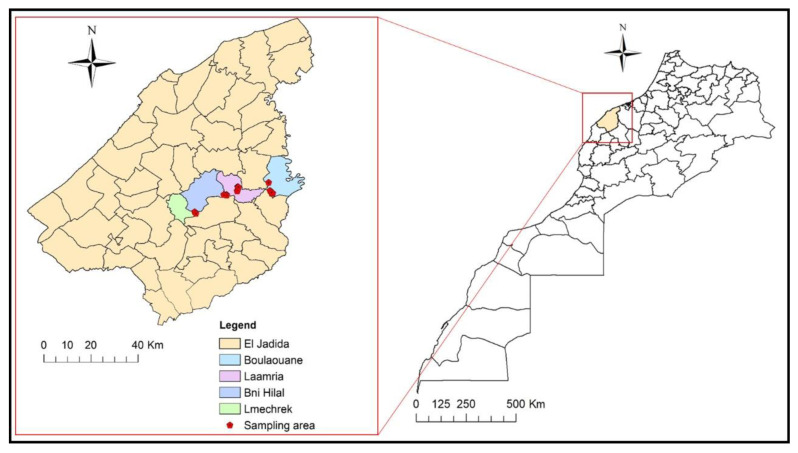
Map showing locations of the Doukkala-Abda region in Morocco (**right square**) and enlarged views of Boulaouane, Laamria, Bni Hilal and Lmechrek (**left square**). The map was created by ArcGIS software version 10.9 (Redlands, CA, USA) and indicates the names of different districts where samples were collected.

In 2021, surveys were conducted in twenty vineyards in Doukkala-Morocco (Figure 6). The sampling was designed under regional viticultural organizations such as the Regional Directorate of Agriculture (DRA), the Provincial Directorate of Agriculture (DPA) and actors involved in the vine’s commercial circuit at the regional level. 

The studied vineyards are spread out over the province to capture possible the heterogeneity that exists in the field as much as possible (Table 2). This area is characterized by a very diverse varietal profile, namely: Muscat of Italy, Red Globe, Victoria, Early Sweet, Doukkali, Aadari and Abou, ranging between 2 and 20 years. The surveyed vineyards correspond to a density of 3333.33 plants per hectare.

The criteria by which the target areas were selected were early symptoms of vine decline such as reduced growth and vigor, leaf chlorosis, or other more obvious symptoms such as wilting, yellowing, defoliation and dieback of shoots and branches.

### 4.2. Fungal Isolation

For isolation, wood segments with cankers were cut from the affected branches and washed under running tap water to remove soil particles. Samples were then transported to the laboratory in separate plastic bags and stored at 4 °C until cultures were isolated. Afterwards, logs (small portions of wood approximately 2 mm^3^) were cut with a sterile scalpel, sterilized in a sodium hypochlorite solution (NaOCl, 2%) for 60 s, rinsed twice using sterile distilled water (SDW), deposited on sterilized Whatman filter paper and air-dried in a laminar flow hood. Three wood pieces were subsequently placed onto a Petri dish (9 cm in diameter) containing 20 mL of potato dextrose agar (PDA, Merck, Darmstadt, Germany) supplemented with 50 mg streptomycin sulfate/L [28,29,30,31]. All plates were incubated in the dark at 25 ± 1 °C for at least 4–7 days. Then, fungal colonies similar to known wood disease fungi were individually purified and transferred to fresh PDA plates for further morphological and molecular characterization.

### 4.3. Pathogenicity Test

To fulfill Koch’s postulates and to ensure that all fungal isolates were able to produce the disease, a pathogenicity test was performed on grapevine-detached young shoots (Growth stage 1: leaf development/BBCH scale).

Green immature shoots of *Vitis vinifera* L. (about 30 cm long) were inoculated with the representative isolate. The shoots were surface sterilized by dipping in 70% ethanol, rinsed in SDW and then damp-dried on sterile paper tissues and dried in a laminar flow hood. Wounds were made in the center of each twig (6 mm length). Each wound was inoculated with a mycelial agar plug from the edge of an actively 10-days growing colony of the fungus grown on a PDA medium. Inoculated wounds were sealed with Parafilm. In addition, the inoculated branches were maintained in an upright position with their lower ends submerged in 1 L jars with 500 mL of SDW in a growth chamber at 23 °C with a 12-h photoperiod (12 h light/12 h dark). The shoots were covered with a plastic bag for the first 4 days to keep the environment moist. Ten branches were used, while plugs of PDA without fungus served as controls. The water was changed every 3 days [32]. The experiment was repeated twice over time. The lengths of the lesion were measured 15 days after inoculation.

### 4.4. Molecular Identification of Isolates

To confirm the exact identity of this fungus, DNA extraction was carried out following the protocol described by Doyle and Doyle [33]. A small amount of mycelium (about 1 cm^2^) was scraped from the surface of pure fresh-growing PDA culture and deposited into an Eppendorf tube (1.5 mL), followed by a 500 μL extraction buffer. The mixture was ground with a pestle, lightly vortexed and incubated for 30 min at 65 °C in a warm-water bath before being briefly centrifuged at 13,000 rpm for 5 min. An equivalent volume (400 μL) of chloroform/isoamyl alcohol (24 v/1 v) was added to the supernatant (400 μL). The obtained mixture was mixed thoroughly for 5 min before being centrifuged at 14,000 rpm for 5 min. The supernatant (350 μL) was then collected and precipitated with 350 μL of isopropanol. The tubes were once again centrifuged at 14,000 rpm for 10 min. After discarding the supernatant, 500 μL of 70% ethanol was added to the pellet, which was vortexed and centrifuged for 5 min at 14,000 rpm. The resulting pellet (DNA) was dried in an incubator at 60 °C for 30 to 45 min before being resuspended in 50 μL SDW and preserved at −20 °C for later use. 

A polymerase chain reaction (PCR) assay was used to detect the representative isolate. Amplification of the internal transcribed spacer region was performed using universal primers ITS1/ITS4 [34], Bt2a/Bt2b [35] and CAL-228F/CAL-737R [19]. Reaction ingredients included 2.5 μL PCR buffer (10 mM), 2.25 μL MgCl2 (50 mM), 0.25 μL dNTPs (10 mM), 1 µL of each primer (10 µΜ), 0.2 µL of Dream Taq DNA Polymerase (5 U/μL) (Thermo scientific, Waltham, MA, USA) and 2.5 μL of genomic DNA; the mixture was then completed with 15.3 μL SDW to reach a final 25 μL volume. The PCR amplifications were carried out using an Eppendorf thermal cycler.

Afterwards, the PCR products were then sequenced. BioEdit software version 7.0.5.3 (Raleigh, NC, USA) was used to edit and align the forward and reverse ITS, β-tubulin and cmdA sequences. The resulting sequences were compared to similar sequences in GenBank databases (National Center for Biotechnology Information) (https://blast.ncbi.nlm.nih.gov/Blast.cgi accessed on 11 December 2021) using Blast search, and then submitted to GenBank. Phylogenetic analysis was performed using MEGA 11.0.8 software (Philadelphia, PA, USA). The acquired ITS, β-tubulin and calmodulin sequences were used to create a phylogenetic tree and isolates were rearranged to form major clusters. Phylogenetic relationships were estimated using the maximum likelihood (ML) method. 1000 bootstrap replications were used to assess the support for each branch in the inferred tree.

### 4.5. Morphological Identification of the Pathogen

To induce the fungus to sporulate, the protocol of Berraf-Tebbai et al. [36] was adopted with slight modifications. Cultures were transferred to PDA with double-autoclaved pine needles on the agar surface. Plates were incubated at 25 °C under mixed near-UV and cool-white fluorescent light in a 12 h light/12 h dark regime for 2–6 weeks [36]. The length and width of 100 conidia from the representative isolate were measured and recorded with the aid of a compound microscope (Olympus EX41) with a ToupView.Ink camera. The mean and standard deviation of the conidial measurements were calculated.

### 4.6. Physiological Traits

#### 4.6.1. Effect of Temperature on Radial Growth Rate

*L. viticola* was subcultured on PDA medium (PDA, Merck, Darmstadt, Germany) at 25 ± 1 °C for 7 days in darkness. 6 mm mycelial agar plugs were cut from one-week-old *Lasiodiplodia* colonies using a cork borer and were aseptically transferred to the center of a 90 mm petri dish containing 20 mL of PDA.

For assessing the effects of temperature on the daily radial growth, the experiment was conducted in incubators equipped with 75 Wm^−2^ cool white light Memmert, IN30 (Schwabach, Germany) (16 h light/8 h dark) and 80% relative humidity at 5, 10, 15, 20, 25, 30, 35 and 40 °C, with four repetitions per treatment. Starting on the second day after inoculation, colony size was measured daily for 20 days on two perpendicular axes for each plate using a digital caliper. The experiment was conducted twice over time with three replicates [27,37,38].

#### 4.6.2. Effect of pH on Radial Growth Rate

In the pH studies, PDA medium was modified with a citric acid buffer (0.1 M sodium citrate and 0.1 M HCl) to achieve pH values of 3.0 and 4.0. For pH values ranging between 5.0, 6.0 and 7.0, a phosphate buffer was used, whilst a boric acid–borax buffer was added to the medium to obtain pH values ranging between 8.0 and 12.0 [39,40]. The acidity was checked with a HI8519 BENCH-TOP pH/mV meter Hanna Instruments, SM76 (Woonsocket, RI, USA). Daily growth was determined as previously described and the experiment was repeated twice over time with three replicates.

### 4.7. Statistical Analysis

All tests were carried out using a completely randomized design (CRD) and were repeated twice. All datasets were summarized as mean ± SD (standard deviation). Datasets were square-root transformations to improve normality and stabilize the variance before the analysis of variance (ANOVA). When the effect was revealed to be significant, the Tukey test was performed and the means were determined to be different for values of *p* ≤ 0.05. All analyses were performed using SPSS statistical software version 25, IBM© SPSS Statistics 25 (Chicago, IL, USA).

## 5. Conclusions

The report of *L. viticola* on grapevine in Morocco widens the range of *Botryosphaeriaceae* species involved in GTD. Therefore, the epidemiological and economic relevance of this fungal pathogen as a causal agent of trunk diseases in commercial vineyards in Doukkala-Morocco deserves further investigation. Certainly, results from this study could help to further understand the ecophysiological and environmental requirements for *L. viticola* development and to establish relevant models to predict the effect of environmental conditions. The latter represents a high and low risk for disease development, which may help to understand the conditions that facilitate the development of the disease in the field. Therefore, results of this study can help to develop reliable integrated strategies for GTD management.

## Figures and Tables

**Table 1:** Occurrence and distribution of pathogenic fungus in vineyards in Doukkala-Abda region.

Location	No. Vineyards ^1^	Positive Orchards	
		*Lasiodiplodia viticola*	Disease Prevalence (%)
Bni Hilal	1	1	71.43
	2	1	
	3	1	
	4	-	
	5	1	
	6	1	
	7	-	
Laamria	8	1	60
	9	1	
	10	-	
	11	1	
	12	-	
Boulaouane	13	1	40
	14	-	
	15	1	
	16	-	
	17	-	
Lmechrek	18	-	0
	19	-	
	20	-	
**Total**	**20**	**10**	**50**

^1^ Number of surveyed vineyards.

**Table 2:** Coordinates of the sampling points.

Location	Vineyard Code	Coordinates	
		X	Y
Laamria	1D	32°48′16″ N	8°14′41″ W
	2D	32°49′15″ N	8°14′42″ W
	3D	32°49′14″ N	8°14′41″ W
	4D	32°49′14″ N	8°14′41″ W
	5D	32°49′12″ N	8°14′36″ W
Bni Hilal	6D	32°47′27″ N	8°17′16″ W
	7D	32°47′20″ N	8°17′14″ W
	8D	32°47′28″ N	8°17′55″ W
	9D	32°43′36″ N	8°24′35″ W
	10D	32°43′23″ N	8°24′17″ W
	11D	32°45′28″ N	8°18′55″ W
	12D	32°43′37″ N	8°23′35″ W
Boulaouane	13D	32°48′24″ N	8°07′16″ W
	14D	32°47′45″ N	8°06′54″ W
	15D	32°47′48″ N	8°06′39″ W
	16D	32°48′00″ N	8°06′55″ W
	17D	32°50′12″ N	8°07′37″ W
Lmechrek	18D	32°46′13″ N	8°15′38″ W
	19D	32°45′11″ N	8°15′33″ W
	20D	32°46′09″ N	8°15′32″ W

## Data Availability

The data used for the analyses in this study are available within the article.

## References

[1] Pouzoulet J. (2012). Développement d’une Méthodologie PCR en temps réel pour la Détection et la Quantification *in planta* des Principaux Champignons Pathogènes Associés aux Maladies du bois de la vigne. https://oatao.univ-toulouse.fr/8359/.

[2] Oiv (2021). State of the World Vitivinicultural Sector In 2020.

[3] DRA (2019). La Culture de la Vigne au Maroc.

[4] Travadon R., Lecomte P., Diarra B., Lawrence D.P., Renault D., Ojeda H., Rey P., Baumgartner K. (2016). Grapevine pruning systems and cultivars influence the diversity of wood-colonizing fungi. Fungal Ecol..

[5] Kenfaoui J., Radouane N., Mennani M., Tahiri A., Ghadraoui L.E., Belabess Z., Fontaine F., Hamss H.E., Amiri S., Lahlali R. (2022). A Panoramic View on Grapevine Trunk Diseases Threats: Case of Eutypa Dieback, Botryosphaeria Dieback, and Esca Disease. J. Fungi.

[6] Gramaje D., Urbez-Torres J.R., Sosnowski M.R. (2018). Managing grapevine trunk diseases with respect to etiology and epidemiology: Current strategies and future prospects. Plant Dis..

[7] Bertsch C., Ramírez-Suero M., Magnin-Robert M., Larignon P., Chong J., Abou-Mansour E., Spagnolo A., Clément C., Fontaine F. (2013). Grapevine trunk diseases: Complex and still poorly understood. Plant Pathol..

[8] Lawrence D.P., Travadon R., Nita M., Baumgartner K. (2017). TrunkDiseaseID.org: A molecular database for fast and accurate identification of fungi commonly isolated from grapevine wood. Crop Prot..

[9] Larignon P., Fontaine F., Farine S., Clément C., Bertsch C. (2009). Esca et Black Dead Arm: Deux acteurs majeurs des maladies du bois chez la Vigne. C. R. Biol..

[10] Mugnai L., Graniti A., Surico G. (1999). Esca (Black measles) and brown wood-streaking: Two old and elusive diseases of grapevines. Am. Phytopathol. Soc..

[11] Del Frari G., Cabral A., Nascimento T., Ferreira R.B., Oliveira H. (2019). *Epicoccum layuense* a potential biological control agent of esca-associated fungi in grapevine. PLoS ONE.

[12] Rolshausen P.E., Baumgartner K., Travadon R., Fujiyoshi P., Pouzoulet J., Wilcox W.F. (2014). Identification of *Eutypa* spp. Causing *Eutypa* Dieback of Grapevine in Eastern North America. Plant Dis..

[13] Glawe D.A., Dilley M.A., Moller W.J. (1983). Isolation and identification of *Eutypa armeniacae* from *Malus domestica* in Washington State. Mycotaxon.

[14] Eichmeier A., Pečenka J., Peňázová E., Baránek M., Català-García S., León M., Armengol J., Gramaje D. (2018). High-throughput amplicon sequencing-based analysis of active fungal communities inhabiting grapevine after hot-water treatments reveals unexpectedly high fungal diversity. Fungal Ecol..

[15] Mondello V., Songy A., Battiston E., Pinto C., Coppin C., Trotel-Aziz P., Clément C., Mugnai L., Fontaine F. (2018). Grapevine trunk diseases: A review of fifteen years of trials for their control with chemicals and biocontrol agents. Plant Dis..

[16] Larignon P., Spagnolo A., Bertsch C., Fontaine F. (2015). First report of young grapevine decline caused by *Neofusicoccum parvum* in France. Plant Dis..

[17] Hofstetter V., Buyck B., Croll D., Viret O., Couloux A., Gindro K. (2012). What if esca disease of grapevine were not a fungal disease?. Fungal Divers..

[18] Romanazzi G., Murolo S., Pizzichini L., Nardi S. (2009). Esca in young and mature vineyards, and molecular diagnosis of the associated fungi. Eur. J. Plant Pathol..

[19] Slippers B., Wingfield M.J. (2007). *Botryosphaeriaceae* as endophytes and latent pathogens of woody plants: Diversity, ecology and impact. Fungal Biol. Rev..

[20] Úrbez-Torres J.R., Gubler W.D. (2009). Pathogenicity of *Botryosphaeriaceae* species isolated from grapevine cankers in California. Plant Dis..

[21] Urbez-Torres J.R., Peduto F., Striegler R.K., Urrea-Romero K.E., Rupe J.C., Cartwright R.D., Gubler W.D. (2012). Characterization of fungal pathogens associated with grapevine trunk diseases in Arkansas and Missouri. Fungal Divers..

[22] Cruywagen E.M., Slippers B., Roux J., Wingfield M.J. (2017). Phylogenetic species recognition and hybridisation in *Lasiodiplodia*: A case study on species from baobabs. Fungal Biol..

[23] Marques M.W., Lima N.B., De Morais M.A., Barbosa M.A.G., Souza B.O., Michereff S.J., Phillips A.J.L., Câmara M.P.S. (2013). Species of *Lasiodiplodia* associated with mango in Brazil. Fungal Divers..

[24] Comont G., Mayet V., Marie-France C. (2016). First Report of *Lasiodiplodia viticola*, *Spencermartinsia viticola* and *Diplodia intermedia* Associated With *Vitis vinifera* Grapevine Decline in French Vineyards. Plant Dis..

[25] Saha A., Mandal P., Dasgupta S., Saha D. (2008). Influence of culture media and environmental factors on mycelial growth and sporulation of *Lasiodiplodia theobromae* (Pat.) Griffon and Maubl. J. Environ. Biol..

[26] Úrbez-Torres J.R. (2015). The status of *Botryosphaeriaceae* species infecting grapevines. Phytopathol. Mediterr..

[27] Copes W.E., Fruit S., Hendrix F.F. (2014). Effect of Temperature on Sporulation of *Botryosphaeria dothidea*, *B. obtusa*, and *B. rhodina*. Plant Dis..

[28] Michailides T.J., Morgan D.P. (1993). Spore release by *Botryosphaeria dothidea* in pistachio orchards and disease control by altering the trajectory angle of sprinklers. Phytopathology.

[29] Michailides T.J. (1991). Pathogenicity, distribution, sources of inoculum, and infection courts of *Botryosphaeria dothidea* on pistachio. Phytopathology.

[30] Pusey P.L., Bertrand P.F. (1993). Seasonal infection of nonwounded peach bark by *Botryosphaeria dothidea*. Phytopathology.

[31] Ammad F., Benchabane M., Toumi M., Belkacem N., Guesmi A., Ameur C., Lecomte P., Merah O. (2014). Occurrence of *Botryosphaeriaceae* species associated with grapevine dieback in Algeria. Turk. J. Agric. For..

[32] Kraus C., Voegele R.T., Fischer M. (2019). Temporal Development of the Culturable, Endophytic Fungal Community in Healthy Grapevine Branches and Occurrence of GTD-Associated Fungi. Microb. Ecol..

[33] Bruez E., Larignon P., Compant S., Rey P. (2017). Investigating the durable effect of the hot water treatment used in nurseries on pathogenic fungi inhabiting grapevine wood and involved in Grapevine Trunk Diseases. Crop Prot..

[34] Mondello V., Giambra S., Conigliaro G. (2020). Fungal pathogens associated with grapevine trunk diseases in young vineyards in Sicily. Phytopathol. Mediterr..

[35] León M., Berbegal M., Rodríguez-Reina J.M., Elena G., Abad-Campos P., Ramón-Albalat A., Olmo D., Vicent A., Luque J., Miarnau X. (2020). Identification and characterization of *Diaporthe* spp. associated with twig cankers and shoot blight of almonds in Spain. Agronomy.

[36] Doyle J., Doyle J. (1990). Isolation of plant DNA from fresh tissue. Focus.

[37] Ezrari S., Radouane N., Tahiri A., Amiri S., Lazraq A., Lahlali R. (2021). Environmental Effects of Temperature and Water Potential on Mycelial Growth of *Neocosmospora solani* and *Fusarium* spp. Causing Dry Root Rot of Citrus. Curr. Microbiol..

[38] Frans M., Aerts R., Van Laethem S., Ceusters J. (2017). Environmental effects on growth and sporulation of *Fusarium* spp. causing internal fruit rot in bell pepper. Eur. J. Plant Pathol..

[39] White T.J., Bruns T., Lee S., Taylor J. (1990). Amplification and direct sequencing of fungal ribosomal RNA genes for phylogenetics. PCR Protocols: A Guide to Methods and Applications.

[40] Chen M.Y., Yan D.D., Han D.K., Ma D.C., Gao P.Z., Bao D.X., Wang M.F. (2022). Occurrence of necrosis of balloon flower (*Platycodon grandiflorus*) caused by *Nigrospora sphaerica* in China. Plant Dis..

